# Supplementary material for: Insights into pulmonary phosphate homeostasis and osteoclastogenesis emerge from the study of pulmonary alveolar microlithiasis
Source: Nat Commun. 2023 Mar 2;14:1205. doi: 10.1038/s41467-023-36810-8 (PMC9981730; doi:10.1038/s41467-023-36810-8)
Supplement: Supplementary file 1 — Supplementary Information [file 41467_2023_36810_MOESM1_ESM.pdf]

Supplementary Information

Insights into pulmonary phosphate homeostasis and osteoclastogenesis emerge from the study of pulmonary alveolar microlithiasis

Yasuaki Uehara, Yusuke Tanaka, Shuyang Zhao, Nikolaos M. Nikolaidis, Lori B. Pitstick, Huixing Wu, Jane J. Yu, Erik Zhang, Yoshihiro Hasegawa, John G. Noel, Jason C. Gardner, Elizabeth J. Kopras, Wendy D. Haffey, Kenneth D. Greis, Jinbang Guo, Jason C. Woods, Kathryn A. Wikenheiser-Brokamp, Jennifer E. Kyle, Charles Ansong, Steven L. Teitelbaum, Yoshikazu Inoue, Göksel Altinişik, Yan Xu and Francis X. McCormack

Supplementary Fig. 1, related to Figure 2.

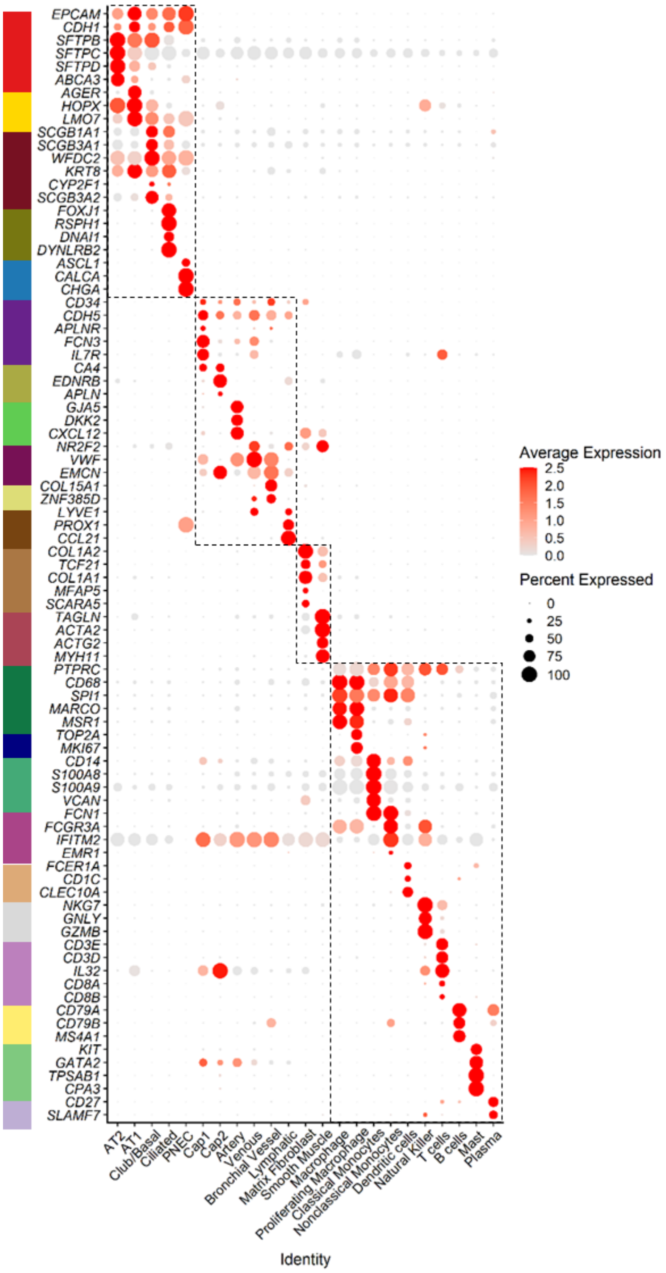

Supplementary Fig. 1 | Integrated Single cell RNA-seq analysis identified diverse cell populations. 23 cellular populations were identified from 14,210 cells from 2 PAM lung samples and 1 control donor lung sample (Fig. 2a). Expression of known cell type markers was used to validate the cell type assignments. Node size represents gene expression frequency in a cell type. Node color gradient represents the average gene expression in a cell type.

Supplementary Fig. 2, related to Figure 2.

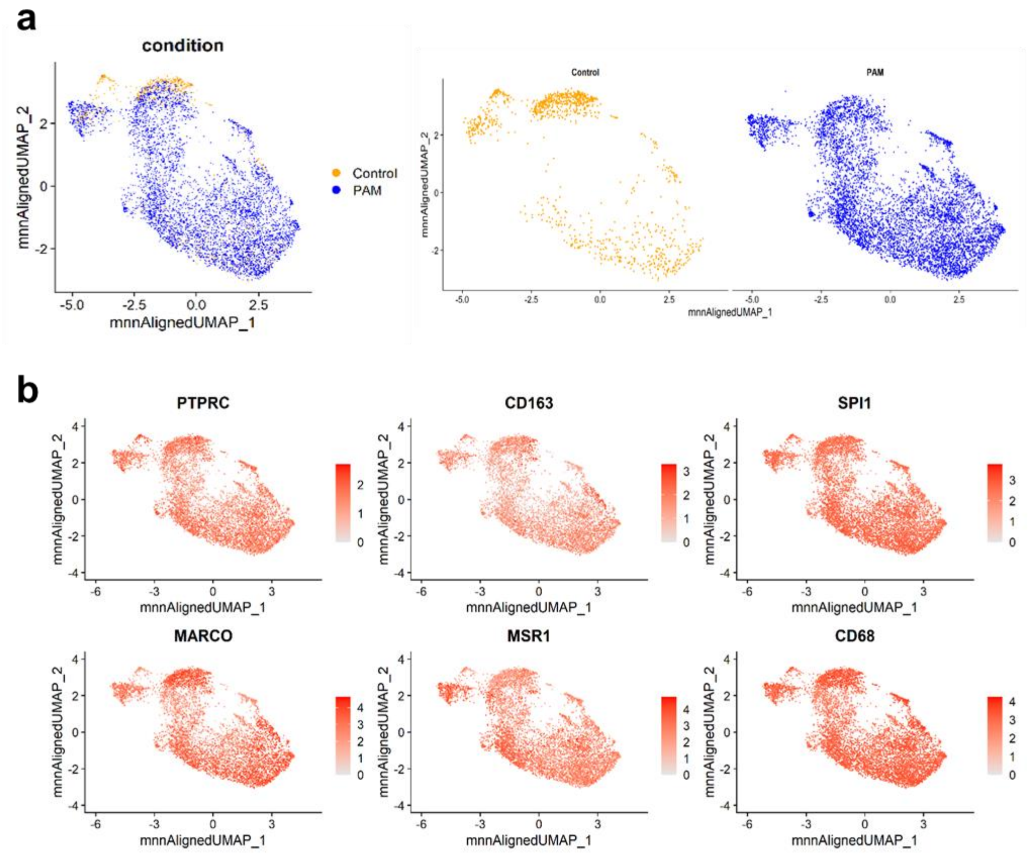

Supplementary Fig. 2 | Integration of PAM macrophage cells and control macrophage cells. **a**. Total of 7,781 macrophage cells from 2 PAM samples and 1 normal control sample are integrated using mutual nearest neighbor (MNN) based batch correction algorithm. UMAP represents the macrophage cells are integrated, and cells are labeled using blue and orange to represent their condition. Left panel shows the integrated UMAP, and the right panel shows the split UMAP of control macrophage (orange) and PAM macrophage (blue). **b**. Expression of selective known macrophage markers is shown via feature plots.

Supplementary Fig. 3, related to Figure 3.

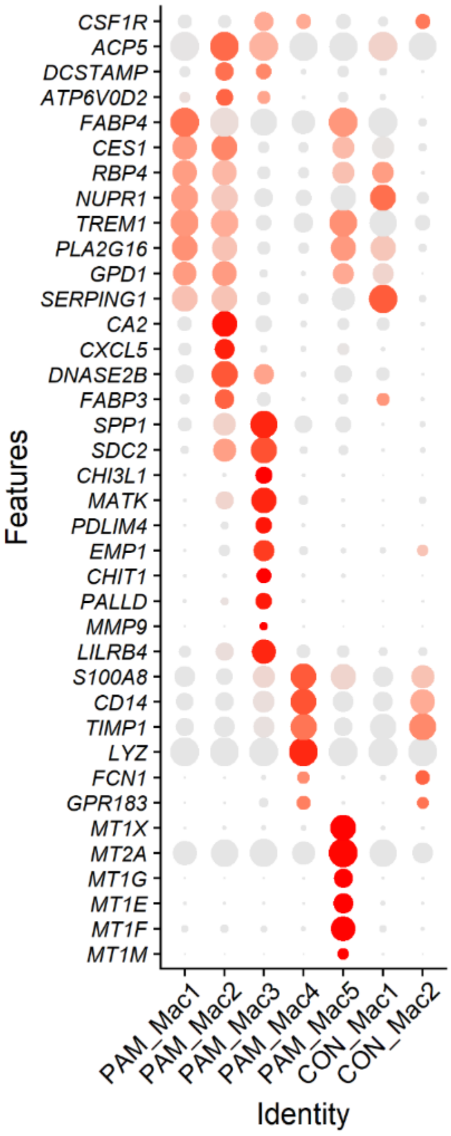

Supplementary Fig. 3 | Macrophages sub-clustering identified heterogeneity within the macrophage populations in PAM and control. scRNA-seq data of 7,781 macrophage cells from 2 PAM samples and 1 control sample were used for this analysis (Fig. 3c). Macrophage cells were clustered using Jaccard-Louvain clustering algorithm. Dot plot showing the expression of top selected differentially expressed genes for each macrophage sub-population. Node size represents gene expression frequency in each cell group. Node color represents the average gene expression in the cell group.

Supplementary Fig. 4, related to Figure 3.

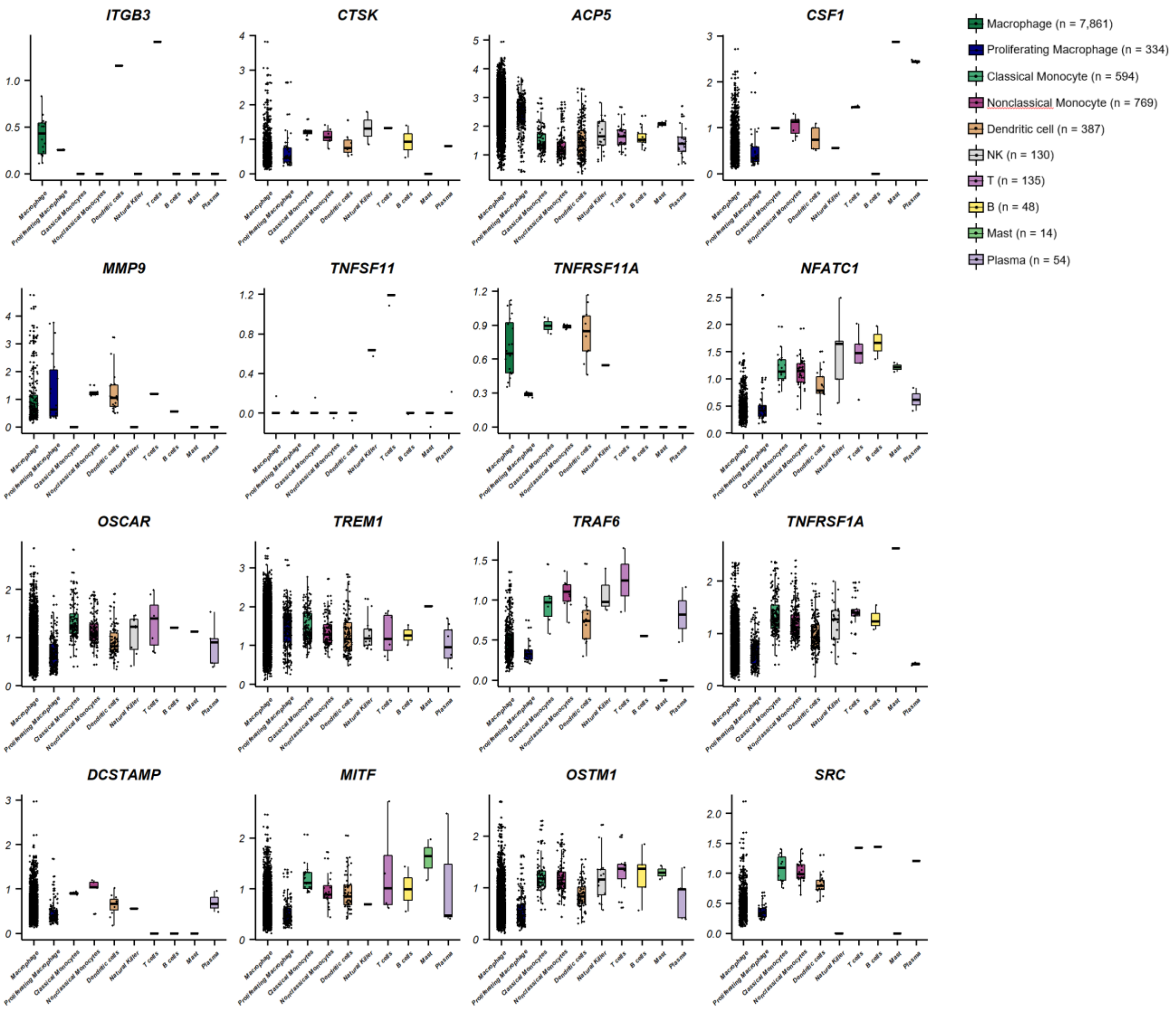

Supplementary Fig. 4 | Expression of RANK (TNFRSF11A) and other osteoclast markers in different immune cells and macrophage subtypes. Boxplots of expression of selected osteoclast marker genes in immune cells (n = 21,075). Figure legend showing the colors representing each cell populations and cell population size. Box center lines, bounds of the box, and whiskers indicate medians, first and third quartiles, and minimum and maximum values within 1.5×IQR (interquartile range) of the box limits, respectively. Significance was determined using a two-sided, unpaired Wilcoxon rank-sum test The expressions are log-normalized.

Supplementary Fig. 5, related to Figure 4.

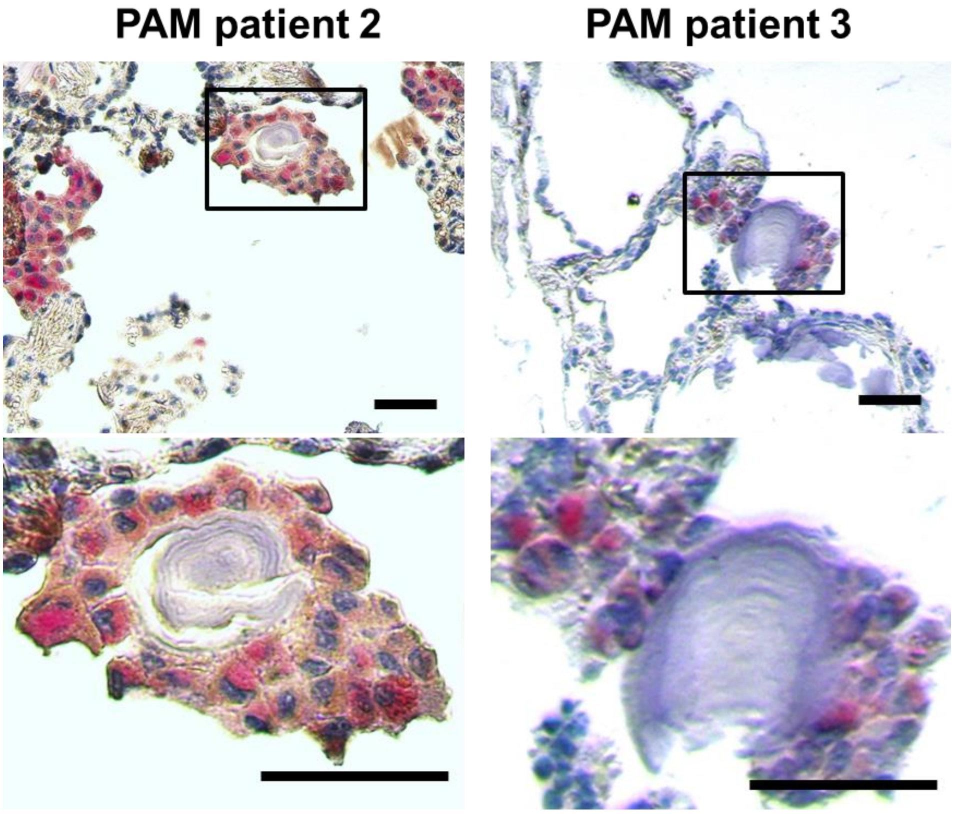

Supplementary Fig. 5 | TRAP-positive multinucleated giant cells in the lungs of other PAM patients. Lung sections from the other PAM patients were fixed and stained for co-staining of TRAP (red) and CALCR (brown) (PAM patient 2) or TRAP (red) and CTSK (brown) (PAM patient 3). Bold scale bar, 50 μm.

Supplementary Fig. 6, related to Figure 5.

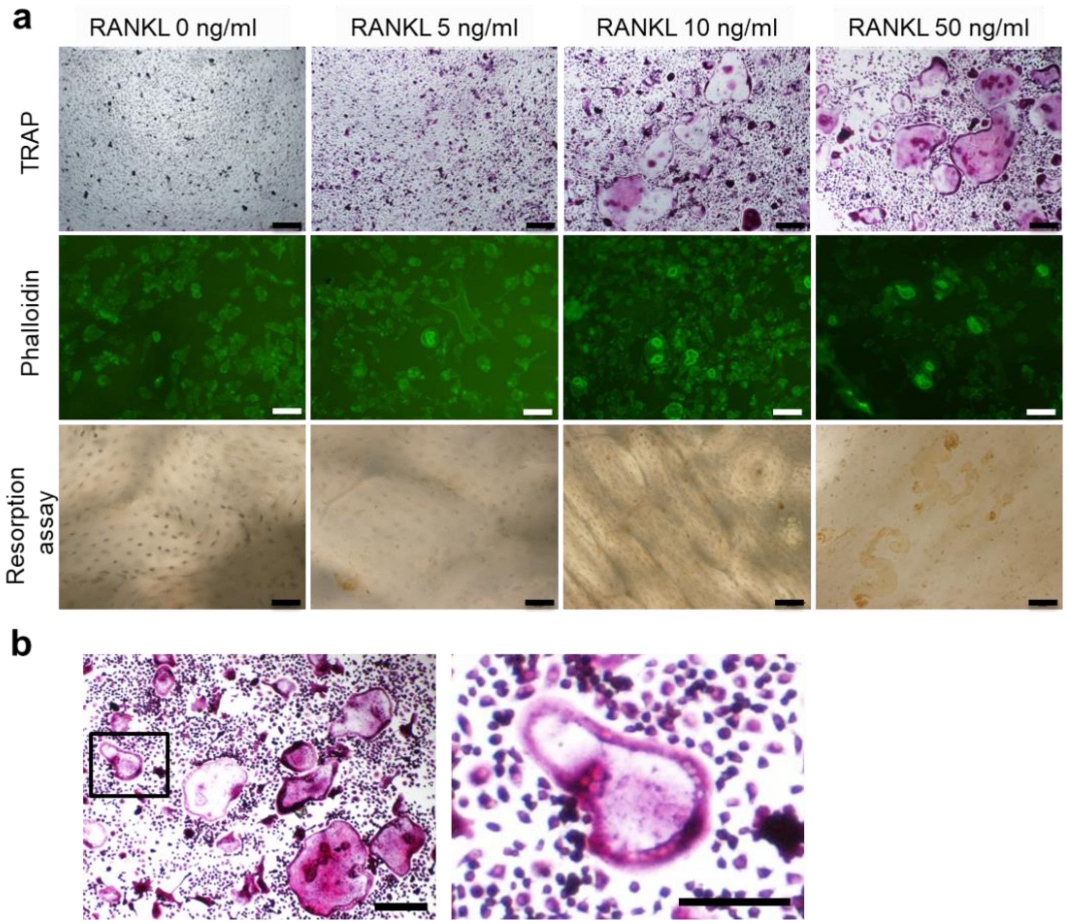

Supplementary Fig. 6 | RANKL induced BMM cell and BAL cell osteoclast differentiation. **a**, BMMs from C57BL/6J mice were cultured in the presence of M-CSF for 4 days on petri dish then re-plated on cell culture dish and incubated with M-CSF ± RANKL for 6 days. After incubation, the cells were stained for TRAP activity (top). For bone resorption assay, BMMs were plated on bovine bone slices and incubated with M-CSF ± RANKL for 6 days. Actin rings were visualized by phalloidin staining (middle) and resorption pits were visualized by peroxidase-conjugated wheat germ agglutinin/horse radish peroxidase staining (bottom). Scale bars: 200 μm (top) and 50 μm (middle and bottom). **b**. BAL cells isolated from CCR2$^{-/-}$ mice were incubated with M-CSF and RANKL for 6 days. After incubation, the cells were stained for TRAP activity. Scale bars: 200 μm.

Supplementary Fig. 7, related to Figure 6.

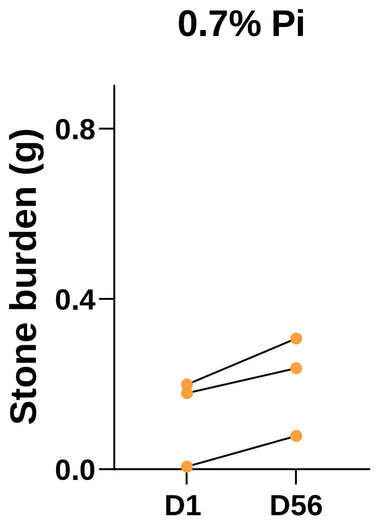

Supplementary Fig. 7 | Microlith volume change after RD diet treatment in 5 to 6 weeks old mice. Stone burden in the lung at day 0 and day 56 calculated from CT images of 5-6 week old mice treated with RD (0.7% Pi). N = 3. Source data are provided as a Source Data file.

Supplementary Fig. 8, related to Figure 6.

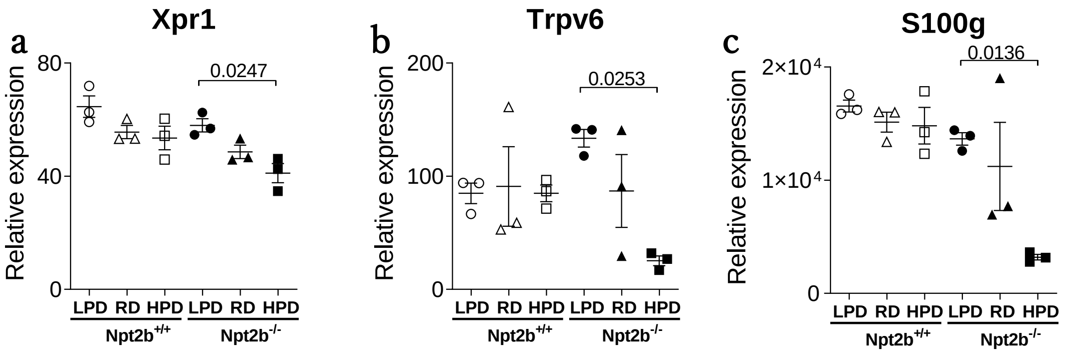

Supplementary Fig. 8 | Dietary phosphate modulates Xpr1 phosphate transporter and VitD3 dependent calcium transporters in Npt2b$^{-/-}$ mice. 5- to 6-week-old Npt2b$^{+/+}$ and Npt2b$^{-/-}$ mice were fed with 0.7% Pi diet for 2 weeks as pretreatment, then they were fed with regular, low or high phosphate diets (0.7% Pi (RD), 0.02% Pi (LPD) or 2% Pi (HPD), respectively) for 1 week. Relative expression of phosphate transporter Xpr1 (**a**), and calcium transporters, Trpv6 (**b**) and S100g (**c**) in the isolated AECII cells from Npt2b$^{+/+}$ and Npt2b$^{-/-}$ mice fed with each indicated diet were determined by RT-PCR. *n* = 4 mice per group. Data are expressed as means ± SD. P values shown in charts determined by one-way ANOVA Tukey's multiple comparisons test (**a-c**). Source data are provided as a Source Data file.

Supplementary Fig. 9, related to Figure 7.

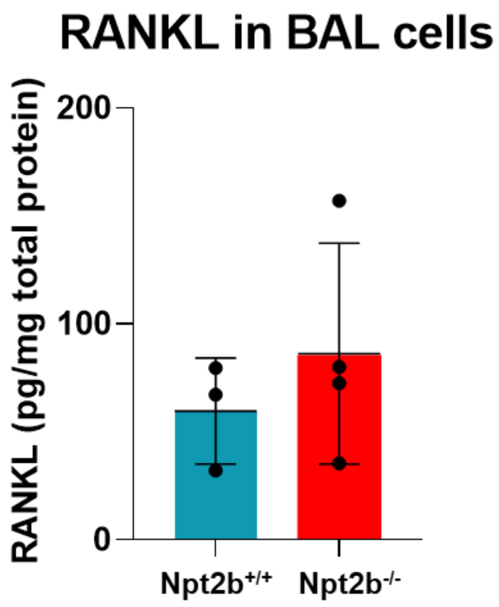

Supplementary Fig. 9 | Expression of RANKL in Npt2b$^{+/+}$ and Npt2b$^{-/-}$ BAL cells. RANKL levels in BAL cell lysate from Npt2b$^{+/+}$ and Npt2b$^{-/-}$ mice were measured by ELISA. n=3 for Npt2b$^{+/+}$ mice and n=4 for Npt2b$^{-/-}$ mice . Data are expressed as means ± SD. Source data are provided as a Source Data file.

Supplementary Table 1. The molar ratios and weight percentages of calcium and phosphorus in microliths.

Microliths	Weight percentage Ca	Weight percentage P	Ca/P Molar ratio
Hydroxyapatite	29.12 ± 6.48	12.62 ± 2.84	2.31 ± 0.06
Mouse microliths	18.98 ± 1.19	9.47 ± 0.71	2.01 ± 0.03
Human microliths	18.50 ± 3.98	9.75 ± 1.40	1.88 ± 0.18

Supplementary Table 2. Lipid species detected in the BALF and microlith isolated from human and Npt2b$^{-/-}$ mice.

		Number of lipids identified		
Subclass		Mouse BALF	Mouse microlith	Human microlith
Carnitine	carnitine	2	0	0
CE	cholesteryl ester	4	5	1
Cer	ceramide	12	6	16
HexCer	hexosylceramide	2	1	1
LacCer	lactosylceramide	1	1	1
SM	sphingomyelin	10	8	14
LPC	lysophosphatidylcholine	0	12	3
PC	phosphatidylcholine	88	80	45
PCox	oxidized PC	1	1	0
PCO	1-O-alkyl-2-acyl-PC	5	6	5
PCP	PC plasmalogen	1	5	7
LPE	lysophosphatidylethanolamine	0	3	4
PE	phosphatidylethanolamine	28	19	16
PEO	1-O-alkyl-2-acyl-PE	3	3	3
PEP	PE plasmalogen	14	15	20
LPG	lysophosphatidylglycerol	4	0	0
PG	phosphatidylglycerol	28	55	21
PI	phosphatidylinositol	1	13	7
PS	phosphatidylserine	0	1	0
MG	Monoacylglycerol	3	0	0
DG	Diacylglycerols	13	11	9
TG	triacylglycerol	20	15	23

Supplementary Table 3. Primer sequences used for the quantitative real time RT-PCR.

Gene	Forward primer	Reverse primer
Acp5	GCCACAGTTATGTTTGTACGTG	ACAGATTGCATACTCTAAGATCTCC
Mmp9	GTGGGAGGTATAGTGGGACA	GACATAGACGGCATCCAGTATC
Ctsk	ATCTCTCTGTACCCTCTGCAT	GACTCTGAAGATGCTTACCCA
Itgb3	ACAGTCATCCTCGTTCTTGTAG	GAACGCTCCATGAAGAAAACAC
Calcr	GGTTTGCCTCATCTTGGTCA	TCTACTACAACGACAACTGCTG
Csf1	GGAAGATGGTAGGAGAGGGTA	AGGATGAGGACAGACAGGT
Tnfrsf11a	AGTGCTGTCTTCTGATATTCTGT	CAGGAGAGGCATTATGAGCAT
Slc20a1	GTGTCCCTTCTCTTCCAGTTC	TGTTGCCGCTTTTGTAGAG
Slc20a2	CCTGCTCTTCCACTTCCTG	TCTTGTGTAACTCCGCCTTG
β-actin	ACCTTCTACAATGAGCTGCG	CTGGATGGCTACGTACATGG
Xpr1	CTGTCACCATCTTCAAGTTCATTG	GGCGAGTGATTCATGTAGAGG
Trpv6	CATGCTTAACCTCCTCATTGC	TTCCGCTCTAACATCACAGTG
S100g	TCAGAGTTCCCCAGCCT	TCCTGACTTGTTCATTGTGAGAG
