# Supplementary material for: Insights into pulmonary phosphate homeostasis and osteoclastogenesis emerge from the study of pulmonary alveolar microlithiasis
Source: Nat Commun. 2023 Mar 2;14:1205. doi: 10.1038/s41467-023-36810-8 (PMC9981730; doi:10.1038/s41467-023-36810-8)
Supplement: Supplementary file 3 — Description of Additional Supplementary Files [file 41467_2023_36810_MOESM3_ESM.docx]

**Description of Additional Supplementary Files**

**Supplementary Data 1.**

**Title:** Proteins identified in human microliths

**Description:** Human microliths were washed, dissolved in EGTA, reduced, alkylated, trypsinized and analyzed by nanoLC-MS/MS, as in Methods. Peptides were identified using homo sapien databases, and ranked according to abundance.

**Supplementary Data 2.**

**Title:** Proteins identified in mouse microliths

**Description:** Mouse microliths were washed, dissolved in EGTA, reduced, alkylated, trypsinized and analyzed by nanoLC-MS/MS, as in Methods. Peptides were identified using mus musculus databases, and ranked according to abundance.

**Supplementary Data 3.**

**Title:** Lipid species identified in mouse and human microliths and mouse bronchoalveolar lavage

**Description:** Total lipid extracts (TLEs) were collected from washed mouse and human microliths and from mouse bronchoalveolar lavage fluid using a modified Folch extraction. Samples were analyzed by mass spectrometry, using LC-MS/MS parameters and identification algorithms outlined in Methods.

**Supplementary Data 4.**

**Title:** Genes differentially expressed in PAM lung macrophages

**Description:** Differentially expressed genes of lung macrophages from a PAM patient (PAM macrophage) vs lung macrophages from a healthy control (control macrophage) were calculated using a binomial based test. Genes with FDR < 0.1, expression frequency > 20%, and the effective size (frequency ratio) > 2 were considered as differentially expressed.
