# Supplementary material for: Insights into pulmonary phosphate homeostasis and osteoclastogenesis emerge from the study of pulmonary alveolar microlithiasis
Source: Nat Commun. 2023 Mar 2;14:1205. doi: 10.1038/s41467-023-36810-8 (PMC9981730; doi:10.1038/s41467-023-36810-8)
Supplement: Supplementary file 8 — Reporting Summary [file 41467_2023_36810_MOESM8_ESM.pdf]

nature portfolio

Corresponding author(s): MCCORMACK AND UEHARA

Last updated by author(s): Nov 29, 2022

Reporting Summary

Nature Portfolio wishes to improve the reproducibility of the work that we publish. This form provides structure for consistency and transparency in reporting. For further information on Nature Portfolio policies, see our Editorial Policies and the Editorial Policy Checklist.

Statistics

For all statistical analyses, confirm that the following items are present in the figure legend, table legend, main text, or Methods section.

n/a	Confirmed	
☐	☒	The exact sample size (*n*) for each experimental group/condition, given as a discrete number and unit of measurement
☐	☒	A statement on whether measurements were taken from distinct samples or whether the same sample was measured repeatedly
☐	☒	The statistical test(s) used AND whether they are one- or two-sided<br>*Only common tests should be described solely by name; describe more complex techniques in the Methods section.*
☒	☐	A description of all covariates tested
☐	☒	A description of any assumptions or corrections, such as tests of normality and adjustment for multiple comparisons
☐	☒	A full description of the statistical parameters including central tendency (e.g. means) or other basic estimates (e.g. regression coefficient) AND variation (e.g. standard deviation) or associated estimates of uncertainty (e.g. confidence intervals)
☐	☒	For null hypothesis testing, the test statistic (e.g. *F*, *t*, *r*) with confidence intervals, effect sizes, degrees of freedom and *P* value noted<br>*Give P values as exact values whenever suitable.*
☒	☐	For Bayesian analysis, information on the choice of priors and Markov chain Monte Carlo settings
☒	☐	For hierarchical and complex designs, identification of the appropriate level for tests and full reporting of outcomes
☒	☐	Estimates of effect sizes (e.g. Cohen's *d*, Pearson's *r*), indicating how they were calculated

Our web collection on statistics for biologists contains articles on many of the points above.

Software and code

Policy information about availability of computer code

Data collection	Cell Ranger R kit version 2.1.1 (10X Genomics) was used to process raw sequencing data and paired-end sequence alignment to the human genome (hg19).
Data analysis	We applied default packages including Seurat 9 (version 2.0 and 3.0), Harmony and Monocle 3 for scRNAseq analysis, all code is publicly available.

For manuscripts utilizing custom algorithms or software that are central to the research but not yet described in published literature, software must be made available to editors and reviewers. We strongly encourage code deposition in a community repository (e.g. GitHub). See the Nature Portfolio guidelines for submitting code & software for further information.

Data

Policy information about availability of data

All manuscripts must include a data availability statement. This statement should provide the following information, where applicable:

- Accession codes, unique identifiers, or web links for publicly available datasets
- A description of any restrictions on data availability
- For clinical datasets or third party data, please ensure that the statement adheres to our policy

scRNAseq data from PAM and donor lungs has been deposited to GEO, accession number: GSE199329 (https://www.ncbi.nlm.nih.gov/geo/).

Human research participants

Policy information about studies involving human research participants and Sex and Gender in Research.

Reporting on sex and gender	There is no clear sex predilection in PAM.<br>The only human tissue same used was from a male child.
Population characteristics	n/a
Recruitment	Serum samples were collected by clinicians caring for PAM patients in the US, Turkey, and Japan. The PAM child was transplanted at the University of Cincinnati and was known to us
Ethics oversight	Human-Institutional Review Board at The University of Cincinnati College of Medicine (#2013-8157)

Note that full information on the approval of the study protocol must also be provided in the manuscript.

Field-specific reporting

Please select the one below that is the best fit for your research. If you are not sure, read the appropriate sections before making your selection.

- [x] Life sciences
- [ ] Behavioural & social sciences
- [ ] Ecological, evolutionary & environmental sciences

For a reference copy of the document with all sections, see nature.com/documents/nr-reporting-summary-flat.pdf

Life sciences study design

All studies must disclose on these points even when the disclosure is negative.

Sample size	Sample size was determined by preliminary experiments. For in vivo studies, based on our preliminary data in similar approaches, we studied 6 animals per group in most experiments (equally divided between males and females)/group to achieve 80% power with a 2-tailed p value of 0.05. For in vitro studies, no sample size calculations were performed. The sample size of each experiment is provided in the figure legends in main manuscript and supplementary information files. These numbers were sufficient for conducting statistical analysis.
Data exclusions	There were no data exclusions
Replication	To assure scientific rigor and reproducible unbiased data, each analysis of cells or tissues was performed performed in triplicate, and repeated at least three times. Animal experiments were replicated at least twice. All replication attempts were successful.
Randomization	All cells were randomly assigned to experimental group. All animals were randomly selected for each group by age and body weight.
Blinding	The counting of TRAP positive cells in Fig 7 was done by a blinded observer. The other experiments and analyses were performed in a non-blinded fashion since the same investigators performed group allocations during data collection and analysis.

Reporting for specific materials, systems and methods

We require information from authors about some types of materials, experimental systems and methods used in many studies. Here, indicate whether each material, system or method listed is relevant to your study. If you are not sure if a list item applies to your research, read the appropriate section before selecting a response.

Materials & experimental systems

n/a	Involved in the study	
[ ]	[x]	Antibodies
[x]	[ ]	Eukaryotic cell lines
[x]	[ ]	Palaeontology and archaeology
[ ]	[x]	Animals and other organisms
[x]	[ ]	Clinical data
[x]	[ ]	Dual use research of concern

Methods

n/a	Involved in the study	
[x]	[ ]	ChIP-seq
[x]	[ ]	Flow cytometry
[x]	[ ]	MRI-based neuroimaging

Antibodies

Antibodies used	Specific primary antibodies for IHC included anti-OPN (ab8448, polyclonal, 1:7500) and anti-cathepsin K (ab19027, polyclonal, 1:750) obtained from Abcam (Cambridge, UK), anti-CALCR LS (LS-A769, polyclonal, 9ug/ml) obtained from LS Bio (Seattle, WA) followed by horseradish peroxidase linked anti-rabbit secondary antibody (#7074, Cell Signaling Technology Inc., Beverly, MA). For in vivo blocking studies, an anti-mouse RANKL mAb (clone IK22-5, Bio X Cell, West Lebanon, NH) or a Rat IgG2a isotype control IgG (clone 2A3, Bio X Cell) was used.<br>For isolation of mouse AT2 cells, CD45 (553076, monoclonal, 1:100) and CD32/16 (553142, monoclonal, 1:100) antibodies (BD Biosciences, San Jose, CA) were used.
Validation	1. Anti-OPN (ab8448, Rabbit polyclonal, 1:7500). Species reactivity: Human. Application: IHC-P. https://www.abcam.co.jp/osteopontin-antibody-ab8448.html<br>2. Anti-cathepsin K (ab19027, Rabbit polyclonal, 1:750). Species reactivity: Mouse, Rat, Human, Zebrafish. Application: IHC-P, ICC/IF, IHC-Fr, WB. https://www.abcam.co.jp/cathepsin-k-antibody-ab19027.html<br>3. Anti-CALCR LS (LS-A769, Rabbit polyclonal, 9ug/ml). Species reactivity: Human, Horse, Rabbit, Application: IHC. https://www.lsbio.com/antibodies/ihc-plus-calcr-antibody-calcitonin-receptor-antibody-transmembrane-domain-ihc-ls-a769/1974?trid=259<br>4. Horseradish peroxidase linked anti-rabbit secondary antibody (#7074, Cell Signaling Technology Inc., Beverly, MA). https://www.cellsignal.jp/products/secondary-antibodies/anti-rabbit-igg-hrp-linked-antibody/7074<br>5. Anti-mouse CD45 (553076, Rat monoclonal, Clone: 30-F11, 1:100). Species reactivity: Mouse. Application: Flow cytometry, Cytotoxicity, IF, IHC, IP. https://www.bdbiosciences.com/ja-jp/products/reagents/functional-cell-based-reagents/purified-rat-anti-mouse-cd45.553076<br>6. Anti-mouse CD16/32 (553142, monoclonal, Clone:2.4G2, 1:100). Species reactivity: Mouse. Application: Blocking, Flow cytometry, IHC, IP. https://www.bdbiosciences.com/ja-jp/products/reagents/flow-cytometry-reagents/research-reagents/single-color-antibodies-ruo/purified-rat-anti-mouse-cd16-cd32-mouse-bd-fc-block.553142<br>7. Anti-mouse RANKL mAb (clone IK22-5, Bio X Cell, West Lebanon, NH)<br>Anti-RANKL antibody was shown to be neutralizing in mice on the manufacturers website (https://bxcell.com/product/m-cd254-trance-rankl/) and in: 1) Lézot F, Chesneau J, Navet B, Gobin B, et al . Skeletal consequences of RANKL-blocking antibody (IK22-5) injections during growth: mouse strain disparities and synergic effect with zoledronic acid. Bone. 2015 Apr;73:51-9. doi:10.1016/j.bone.2014.12.011. Epub 2014 Dec 20. PMID: 25532478.<br>2) Kamijo S, Nakajima A, Ikeda K, et al.Amelioration of bone loss in collagen-induced arthritis by neutralizing anti-RANKL monoclonal antibody. Biochem Biophys Res Commun. 2006 Aug 18;347(1):124-32. doi: 10.1016/j.bbrc.2006.06.098. Epub 2006 Jun 23. PMID: 16815304.<br>8. Rat IgG2a isotype control IgG (clone 2A3, Bio X Cell). https://bioxcell.com/invivomab-rat-igg2a-isotype-control-anti-trinitrophenol-be0089#tab_specifications

Animals and other research organisms

Policy information about studies involving animals; ARRIVE guidelines recommended for reporting animal research, and Sex and Gender in Research

Laboratory animals	C57BL/6J WT mice were obtained from Jackson Laboratories (Bar Harbor, ME). The epithelium-targeted Npt2b-/- mouse model was developed by breeding mice homozygous for floxed Slc34a2 with mice expressing Cre recombinase under the influence of the sonic hedgehog (Shh) promoter, as previously reported. CCR2-/- mice, B6.129S4-Ccr2tm1Ifc/J were purchased from Jackson Laboratories. The age of mice for most experiments was 8-12 weeks, but for those studies requiring young mice we used 5-6 week old mice, and those requiring aged mice, 20-28 weeks or older.
Wild animals	No wild animals were used in the study.
Reporting on sex	Sex was not considered in study design, because there is no clear sex predilection in PAM. Female mice were used in most experiments because male mice, esp. Npt2b mice, fight and kill each other. Male Npt2b mice were used for harvesting microlithss.
Field-collected samples	No field collected samples were used in the study.
Ethics oversight	The University of Cincinnati Institutional Animal Care and Use Committee approved the protocol

Note that full information on the approval of the study protocol must also be provided in the manuscript.
